# Hyperferritinemia as a Diagnostic Marker for Severe Fever with Thrombocytopenia Syndrome

**DOI:** 10.1155/2017/6727184

**Published:** 2017-02-28

**Authors:** Uh Jin Kim, Tae hoon Oh, Bansuk Kim, Seong Eun Kim, Seung-Ji Kang, Kyung-Hwa Park, Sook-In Jung, Hee-Chang Jang

**Affiliations:** Department of Infectious Diseases, Chonnam National University Medical School, Gwangju, Republic of Korea

## Abstract

Severe fever with thrombocytopenia syndrome (SFTS) is an emerging viral disease in East Asia with high mortality. Few studies have examined markers that suggest SFTS in febrile patients. To determine useful biochemical markers for SFTS, patients aged 18 years or older with SFTS or microbiologically confirmed community-onset bacteremia with thrombocytopenia (BT) at presentation between June 2013 and December 2015 were included from two tertiary university hospitals in Republic of Korea retrospectively. Eleven patients with SFTS and 62 patients with bacteremia and thrombocytopenia were identified in the study period. Age and sex did not show significant difference among two groups. Fever was more commonly observed but comorbidities were less common in SFTS than in BT (*P* < 0.05, each). The areas under the curves of serum ferritin, C-reactive protein, white blood cell count, serum procalcitonin, and fibrinogen were above 0.9, indicating the discriminative power of these biomarkers (1.000, 0.991, 0.963, 0.931, and 0.934, resp., all *P* < 0.05). The optimal cutoff value of serum ferritin was 3,822 ng/mL in this study. These results suggest that hyperferritinemia is a typical laboratory feature of SFTS, and the serum ferritin level can be used as a marker for clinicians suspecting SFTS.

## 1. Introduction

Severe fever with thrombocytopenia syndrome (SFTS) is an emerging hemorrhagic fever caused by the SFTS virus of the family Bunyaviridae [1]. The disease was first reported from China and has been recognized in Japan and Korea as well [2, 3]. The mortality ranges from 6.3 to 30% [4]. An early diagnosis of SFTS is important for patient survival and to prevent transmission due to rapid clinical deterioration and high mortality [5]. However, the clinical manifestations of SFTS are nonspecific and include an abrupt fever, fatigue, lack of appetite, and respiratory, gastrointestinal, and neurological symptoms; these are usually accompanied by thrombocytopenia [4]. Although a real-time reverse-transcriptase polymerase chain reaction (RT-PCR) is specific in the diagnosis of SFTS [6], the primary healthcare or emergency clinician must initially suspect SFTS to run the test. Therefore, a surrogate marker for SFTS is needed to differentiate SFTS from sepsis caused by various other infectious agents to help clinicians to decide to request RT-PCR tests for SFTS.

We searched for biomarkers that can differentiate SFTS from bacterial sepsis with thrombocytopenia, which is encountered more frequently in outpatient or emergency settings. We compared the clinical and laboratory findings between SFTS and bacteremia with thrombocytopenia (BT) to identify useful surrogate markers that can differentiate these diseases when SFTS is suspected before performing an SFTS-specific confirmatory test.

## 2. Patients and Methods

### 2.1. Ethics

This study was approved by the Institutional Review Board (IRB) of Chonnam National University Hospital (IRB number CNUHH-2016-021). The IRB granted a waiver of consent given the retrospective nature of the project.

### 2.2. Study Design and Patients

The study included patients aged 18 years or older with SFTS or microbiologically confirmed community-onset bacteremia with thrombocytopenia (BT) at presentation between June 2013 and December 2015 at two tertiary university hospitals in Korea: Chonnam National University Hospital (900 beds) and Chonnam National University Hwasun Hospital (600 beds). Data on age, gender, residence, medical history, clinical manifestations on admission, and results of laboratory tests were collected in a retrospective review of the Electronic Medical Records. The white blood cell (WBC) count, neutrophil count, hemoglobin, platelet count, prothrombin time (PT), activated partial thromboplastin time (aPTT), fibrinogen, albumin, aspartate aminotransferase (AST), alanine aminotransferase (ALT), total bilirubin, C-reactive protein (CRP), lactate dehydrogenase (LDH), and creatine kinase (CK) all obtained within 24 h of hospitalization were collected. In addition, the serum ferritin and procalcitonin (PCT) level within 4 days of hospitalization were obtained.

### 2.3. Definitions

SFTS was defined as confirmation of the SFTS virus with real-time RT-PCR (DiaStar™, SolGent, Daejung, Korea) of a blood sample performed at one of two government institutions: Jeollanam-do Institute of Health and Environment (Muan, Korea) and the Gwangju Institute of Health and Environment (Gwangju, Korea). Community-onset bacteremia was defined as present when microorganisms were isolated from blood cultures within 48 h after admission. Thrombocytopenia was defined as a blood platelet count ≤ 130,000/mm^2^.

### 2.4. Statistical Analyses

Categorical variables were compared using Fisher's exact test, and continuous variables were compared using Mann–Whitney *U*-test, as appropriate. Data are presented as medians and interquartile ranges (IQRs) or as numbers and percentages. The most useful marker for the diagnosis of SFTS and cutoff value of the laboratory marker with the best sensitivity and specificity were determined using the area under the receiver operating characteristic (ROC) curve (AUC). All significance tests were two-tailed, and *P* values ≤ 0.05 indicated statistical significance. The statistical analyses were performed with SPSS ver. 22.0 (IBM, Armonk, NY, USA).

## 3. Results

From June 2013 to December 2015, eleven patients with SFTS and 62 patients with community-onset BT were identified. Of the 62 patients of bacteremia, a urinary tract infection (34 and 55%) was the most common primary site, followed by intra-abdominal infection (7, 11%), skin and soft tissue infection (6, 10%), pneumonia (5, 8%), catheter-related infection (4, 6%), infective endocarditis (2, 3%), osteomyelitis (2, 3%), meningitis (1, 2%), and neutropenic fever of unknown primary origin (1, 2%). The microorganisms identified were* Escherichia coli* (22, 35%),* Klebsiella pneumoniae* (12, 19%),* Staphylococcus aureus* (11, 18%),* Acinetobacter* species (4, 6%),* Enterobacter* species (4, 6%),* Proteus mirabilis* (2, 3%),* Pseudomonas aeruginosa* (2, 3%),* Aeromonas hydrophila* (1, 2%),* Klebsiella oxytoca* (1, 2%),* Serratia marcescens* (1, 2%),* Staphylococcus epidermidis* (1, 2%), and* Streptococcus pneumoniae* (1, 2%).

**Figure 1:**
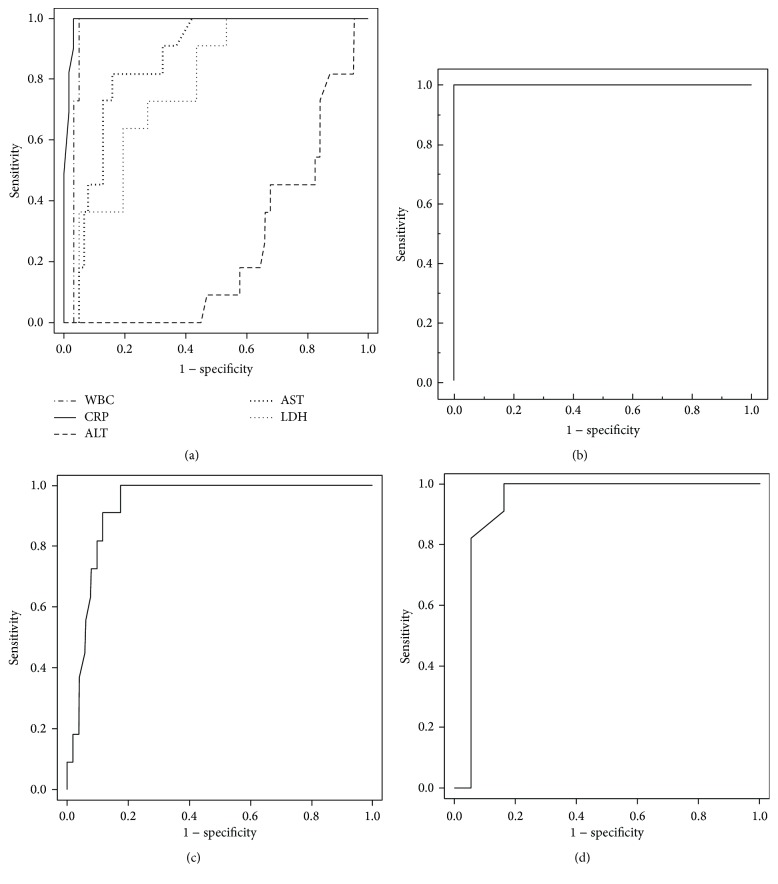
(a) ROC curves of laboratory markers as predictors of SFTS in 73 patients with SFTS or community-onset bacteremia with thrombocytopenia. AUC: CRP 0.991 (95% CI, 0.975–1.000); WBC 0.963 (95% CI, 0.918–1.000); AST 0.857 (95% CI, 0.764–0.950); LDH 0.777 (95% CI, 0.653–0.901); ALT 0.246 (95% CI 0.126–0.367) (all *P* < 0.05). (b) ROC curve of serum ferritin as a predictor of SFTS. *n* = 72 (SFTS 10, bacteremia 62). AUC 1.000 (95% CI, 1.000-1.000) (*P* < 0.001). (c) ROC curve of serum procalcitonin as a predictor for SFTS. *n* = 48 (SFTS 11, bacteremia 37). AUC 0.931 (95% CI, 0.855–1.000) (*P* < 0.001). (d) ROC curve of serum fibrinogen as a predictor for SFTS. *n* = 63 (SFTS 11, bacteremia 52). AUC 0.934 (95% CI, 0.873–0.994) (*P* < 0.001). ROC, receiver operating characteristic; SFTS, severe fever with thrombocytopenia syndrome; WBC, white blood cell count; AST, aspartate aminotransferase; LDH, lactate dehydrogenase; ALT, alanine aminotransferase; CRP, C-reactive protein.

**Figure 2:**
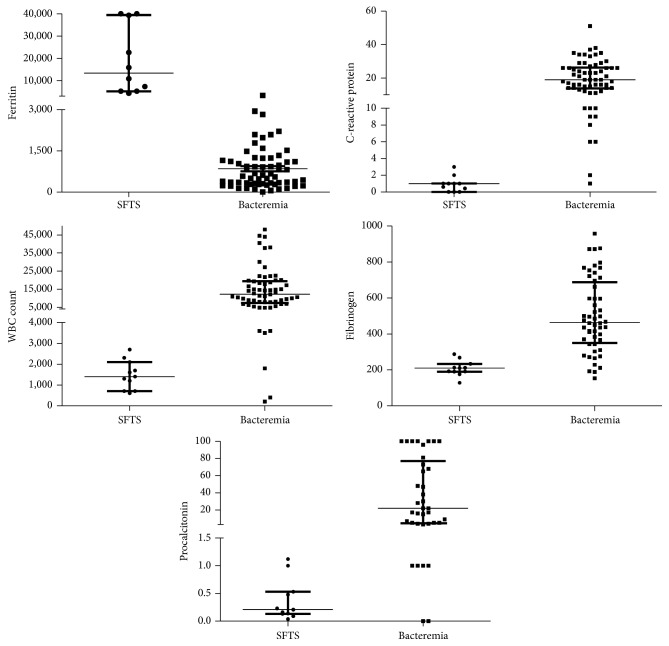
Comparison of serum biomarkers in patients with severe fever with thrombocytopenia syndrome and bacteremia with thrombocytopenia.

The clinical characteristics of both the SFTS and BT groups are summarized in Table 1. The groups did not differ in age or gender. Comorbidities were less common in the SFTS group than in the BT group (5/11* versus* 59/62; *P* < 0.001). The mortality rate was 37% (4/11) in the SFTS group and 29% (18/62) in the BT group (*P* = 0.724). Regarding the laboratory findings, WBC count, CRP, PCT, and fibrinogen were significantly lower in SFTS than BT, while CK, AST, ALT, LDH, aPTT, and ferritin were significantly higher in SFTS than BT (all *P* < 0.05; Table 1). The ROC curve was used to analyze the diagnostic accuracy of biomarkers that differed significantly between the groups (Figure 1). The AUCs of serum ferritin, C-reactive protein, WBC count, serum PCT, and fibrinogen were above 0.9, indicating the discriminative power of the biomarkers (1.000, 0.991, 0.963, 0.931, and 0.934, resp., all *P* < 0.05). The optimal cutoff value of ferritin was 3,822 ng/mL, with a sensitivity of 100% and specificity of 100%.  Figure 2 shows the levels and distributions of these five biomarkers in each patient group.

## 4. Discussion

SFTS is a viral disease that can be diagnosed with SFTS virus PCR tests when suspected clinically. Although fever and thrombocytopenia are the main findings of the disease, these are nonspecific. Therefore, useful surrogate laboratory markers are needed.

Studies have shown that AST, ALT, LDH, and CK are elevated in patients with SFTS [4] and high levels of AST, ALT, LDH, CK, and prolonged aPTT are significant predictors of mortality [7]. In our study, AST, ALT, LDH, and CK were elevated more significantly in SFTS than in BT. However, their usefulness at differentiating SFTS from BT was limited due to the low range of AUC (0.763–0.823) compared with other biomarkers. WBC, CRP, and PCT are useful markers of bacterial sepsis compared with viral diseases and other inflammatory conditions [8, 9]. In SFTS, data on PCT are sparse, while several studies have observed a decreased WBC and neutrophils and no increase in CRP in SFTS [10, 11]. We found that the WBC, CRP, and PCT were useful for differentiating SFTS from BT (all AUC > 0.950). Coagulation disturbances, such as an increased thrombin time, and aPTT have also been reported in patients with SFTS [12, 13]. However, no comparative study with bacterial sepsis has been reported. We also observed prolongation of aPTT in SFTS, and the fibrinogen level was useful for distinguishing SFTS from BT (AUC 0.941).

An elevated serum ferritin is a marker of hemophagocytic lymphocytosis histiocytosis (HLH), macrophage activation syndrome (MAS) in rheumatic disorders, iron-overloaded states including chronic blood transfusions, and acute liver injury [14–16]. However, there are limited data on its clinical use for differentiating among infectious diseases [17, 18]. For infectious diseases, highly elevated serum ferritin has been reported only in several hemorrhagic viral infections, including Dengue [19] and Chikungunya [15] fevers, and viral hepatitis causing liver injury [20]. Although the serum ferritin is elevated as an acute phase reactant in acute extracellular bacterial sepsis, the level is usually not high, because extracellular bacteria trigger hypoferritinemia [21]. We found that the serum ferritin level was elevated markedly in all of the patients with SFTS. The major source of extreme hyperferritinemia seen in HLH or MAS has been suggested to be activated macrophages in the bone marrow or spleen [22, 23]. There is one report of HLH in a patient with SFTS [24], while other studies did not find HLH [10]. In our study, only three patients from the SFTS group underwent a bone marrow biopsy. In one patient, whose initial ferritin level was 13740 ng/mL, presence of hemophagocytosis was found in the bone marrow. A Recent animal SFTS virus infection model study suggested that SFTS virus-induced thrombocytopenia is caused by the clearance of circulating virus-bound platelets by activated splenic macrophages [25]. These findings suggest the possibility of SFTS as one of the infectious causes for HLH and based on animal study, activated macrophages in the spleen might exhibit hemophagocytic activities which may lead to hyperferritinemia. However, recently Kimura et al. showed that* Mycobacteria tuberculosis*, an intracellular pathogen, can induce high-level secretion of ferritin by unconventional activation of secretory autophagy in human monocytes (THP-1 cells), compared to LPS treating in THP1 cells [26]. These findings suggest that serum ferritin can be increased by intracellular infection itself, irregardless of hemophagocytosis. Additional humans studies are needed to determine the exact source and mechanism of hyperferritinemia in SFTS.

This study had several limitations. First, due to its retrospective nature, some data on procalcitonin and fibrinogen are missing from both groups. Second, the clinical and laboratory findings of SFTS were compared only with bacterial sepsis; comparisons of SFTS with other viral diseases, intracellular pathogens such as rickettsia, and inflammatory conditions that can accompany thrombocytopenia are needed to ensure the specificity of these biomarkers. Third, we did not perform bone marrow biopsies in all of the SFTS patients and lack specific data on inflammatory cytokines and viral loads, leaving the mechanism of the extraordinary elevation of serum ferritin in SFTS undetermined.

In short, this study suggests that along with classical inflammatory markers including WBC count, CRP, PCT, and fibrinogen, high serum ferritin level can be used as a biochemical marker to lead clinicians suspecting SFTS to perform confirmatory tests for SFTS.

## Figures and Tables

**Table 1:** Comparison of the basal characteristics and laboratory findings in patients with SFTS and bacteremia with thrombocytopenia.

	STFS (*N* = 11, %)	Bacteremia (*N* = 62, %)	*P* value
Demographic data			
Age	74 (64–80)	69 (59–75)	0.257
Male sex	2 (18)	25 (40)	0.195
Rural area resident	9 (82)	26 (42)	0.150
Comorbidities	5 (46)	59 (95)	<0.001
Hypertension	4 (36)	31 (50)	0.404
Diabetes mellitus	3 (27)	24 (39)	0.736
Neurologic disease	0 (0)	13 (21)	0.195
Cardiovascular disease	0 (0)	11 (18)	0.197
Chronic kidney disease	1 (9)	9 (15)	>0.999
Liver cirrhosis	0 (0)	5 (8)	>0.999
Autoimmune disease	0 (0)	3 (5)	>0.999
Dyslipidemia	1 (9)	1 (2)	0.280
Malignancy	1 (9)	1 (2)	0.280
Symptom			
Fever (>38°C)	11 (100)	37 (60)	0.012
Neurologic symptoms^*∗*^	5 (46)	10 (16)	0.041
Mortality	4 (37)	18 (29)	0.724
Laboratory results			
White blood cell (/mm^3^)	1400 (700–2100)	11850 (7625–19375)	<0.001
Hemoglobin (dL)	12 (11-12)	10 (9–12)	0.024
Platelet (×10^3^/mm^3^)	43 (35–71)	70 (47–98)	0.121
PT (sec)	13 (12–15)	13 (12–15)	0.743
aPTT (sec)	59 (51–67)	47 (40–58)	0.035
Fibrinogen (mg/dL)^†^	210 (190–234)	464 (350–688)	<0.001
AST (U/L)	152 (113–448)	47 (23–85)	<0.001
ALT (U/L)	88 (39–100)	26 (13–69)	0.008
LDH (U/L)	1071 (613–2574)	593 (419–873)	0.004
Creatine kinase (IU/L)^†^	1208 (577–2530)	118 (45–308)	<0.001
CRP (mg/dL)	1.0 (0.1–1.1)	18.7 (13.4–26.5)	<0.001
Ferritin (ng/mL)^†^	10508 (3768–26813)	597 (303–1169)	<0.001
Procalcitonin (ng/mL)^†^	0.2 (0.1–0.5)	22.3 (4.6–76.7)	<0.001

Laboratory data are expressed as the median (interquartile range), unless otherwise specified.

^*∗*^Neurological symptoms include altered mentation, motor weakness, and dysarthria.

^†^Number of patients for biomarkers: fibrinogen (SFTS 11, bacteremia 52), Creatinine kinase (SFTS 11, bacteremia 60), procalcitonin (SFTS 11, bacteremia 37), and ferritin (SFTS 10, bacteremia 62).

SFTS, severe fever with thrombocytopenia syndrome; PT, prothrombin time; aPTT, activated partial thromboplastin time; AST, aspartate aminotransferase; ALT, alanine aminotransferase; LDH, lactate dehydrogenase; CRP, C-reactive protein.
